# Emergency surgery for intestinal obstruction caused by gastrointestinal metastasis of malignant melanoma: a case report

**DOI:** 10.1093/jscr/rjag064

**Published:** 2026-06-26

**Authors:** Daniel Lopez Zertuche, Jessica J Betancourt Ferreyra, Aranza J Sanchez Lopez, Miren L Cardenas Hernandez, Maria Estrada Hernandez, Milca N Deance Rodriguez, Laura M Hernandez Regino

**Affiliations:** Department of Surgery, Dr. Manuel Gea González General Hospital, Calzada de Tlalpan 4800, Col. Sección XVI, Tlalpan, Mexico City 14080, Mexico; Department of Surgery, Dr. Manuel Gea González General Hospital, Calzada de Tlalpan 4800, Col. Sección XVI, Tlalpan, Mexico City 14080, Mexico; Department of Surgery, Dr. Manuel Gea González General Hospital, Calzada de Tlalpan 4800, Col. Sección XVI, Tlalpan, Mexico City 14080, Mexico; Department of Dermatopathology, Dr. Manuel Gea González General Hospital, Calzada de Tlalpan 4800, Col. Sección XVI, Tlalpan, Mexico City 14080, Mexico; Department of Pathology, Dr. Manuel Gea González General Hospital, Calzada de Tlalpan 4800, Col. Sección XVI, Tlalpan, Mexico City 14080, Mexico; Department of Pathology, Dr. Manuel Gea González General Hospital, Calzada de Tlalpan 4800, Col. Sección XVI, Tlalpan, Mexico City 14080, Mexico; Department of Surgery, Dr. Manuel Gea González General Hospital, Calzada de Tlalpan 4800, Col. Sección XVI, Tlalpan, Mexico City 14080, Mexico

**Keywords:** surgical emergency, melanoma, metastasis, bowel obstruction, intussusception

## Abstract

Malignant melanoma (MM) is the least common yet aggressive skin cancer. The gastrointestinal tract is the most frequent site of distant metastasis, although metastases are rarely detected during life. Adult intussusception is uncommon and is usually secondary to neoplasms, including metastases. We report the case of an adult with a history of cutaneous MM presenting with acute intestinal obstruction. Computed tomography revealed jejunojejunal intussusception, and emergency surgery identified three intraluminal tumors. Histopathology confirmed metastatic melanoma as the lead point. Given the acute presentation, management prioritized surgery over staging studies such as positron emission tomography-computed tomography. This case underscores the diagnostic and therapeutic importance of emergency surgical management of intestinal obstruction caused by metastatic melanoma. It also emphasizes the need to maintain a high index of suspicion for malignancy in adult intussusception, where histopathological confirmation remains critical for establishing a definitive diagnosis and guiding both the surgical resolution of the acute obstruction and an appropriate palliative strategy.

## Introduction

Malignant melanoma (MM) is the least common yet most lethal skin cancer and ranks as the fifth most frequent malignancy worldwide [1–4]. The gastrointestinal (GI) tract is a frequent site of metastasis in disseminated MM, although it is rarely detected in living patients and usually reflects an advanced disease [1, 5]. GI symptoms are often nonspecific; however, intussusception is an uncommon presentation typically associated with intestinal obstruction requiring urgent surgery [6, 7]. Imaging plays a central role in diagnosis and staging, with computed tomography (CT) and positron emission tomography CT-being the most sensitive modalities for detecting GI involvement [8]. Additional tests, such as ultrasonography, barium studies, endoscopy aid diagnosis, and tissue confirmation [1, 5]. Management should be tailored by a multidisciplinary team using endoscopy, surgery, chemotherapy, or immunotherapy alone or in combination [9, 10]. Immunotherapeutic advances have significantly improved survival outcomes [10]. We report a rare case of jejunojejunal intussusception caused by intestinal metastatic melanoma that presented as an acute obstruction requiring emergency surgery [6, 7].

## Case presentation

A 70-year-old male with a history of ulcerated invasive nodular melanoma of the right dorsolumbar region (Breslow thickness 15 mm, Clark level IV; T4bNxM1c0) was diagnosed in 2024 by excisional biopsy at our institution (Fig. 1). Sentinel lymph node mapping was not performed due to institutional limitations. A metastatic lesion in the right eye at the iris was subsequently documented by the Department of Ophthalmology (Fig. 2), corresponding to clinical stage IVC according to the AJCC 8a edition. The patient was referred to a cancer center for targeted therapy and received pembrolizumab (anti PD-1) every 3 weeks for 1 year, which was discontinued due to clinical deterioration, as evidenced by an Eastern Cooperative Oncology Group (ECOG) performance status of 3 and a Karnofsky score of 50.

**Figure 1:**
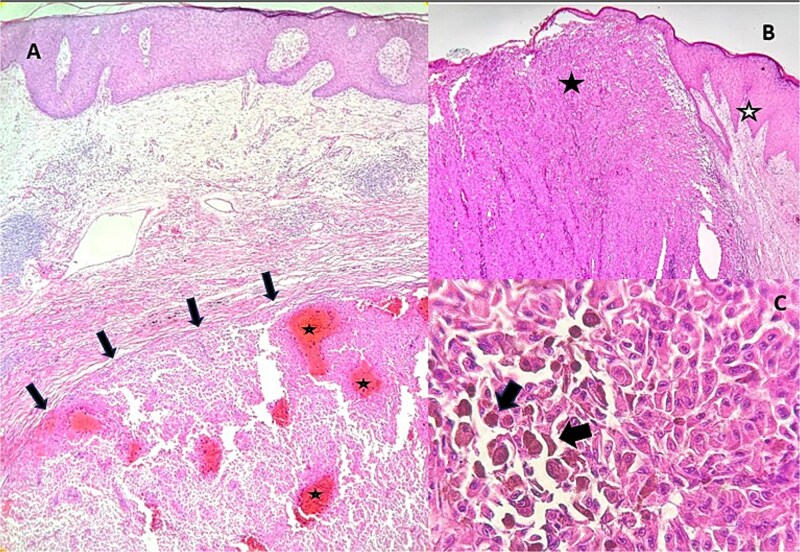
Biopsy of dorsolumbar lesion. (A) Histologic section at 10× magnification showing a nodular dermal neoplasm (black arrows) composed of atypical melanocytes arranged in sheets, with areas of hemorrhage (white stars). (B) Histologic section at 10× magnification showing a dermal neoplasm (black star) that pushes the epidermis (white star), composed of atypical melanocytes arranged in sheets. (C) Histologic section at 40× magnification showing atypical, pleomorphic melanocytes with hyperchromatic nuclei (black arrows), prominent nucleoli, and intensely eosinophilic cytoplasm containing irregular melanin pigment.

**Figure 2:**
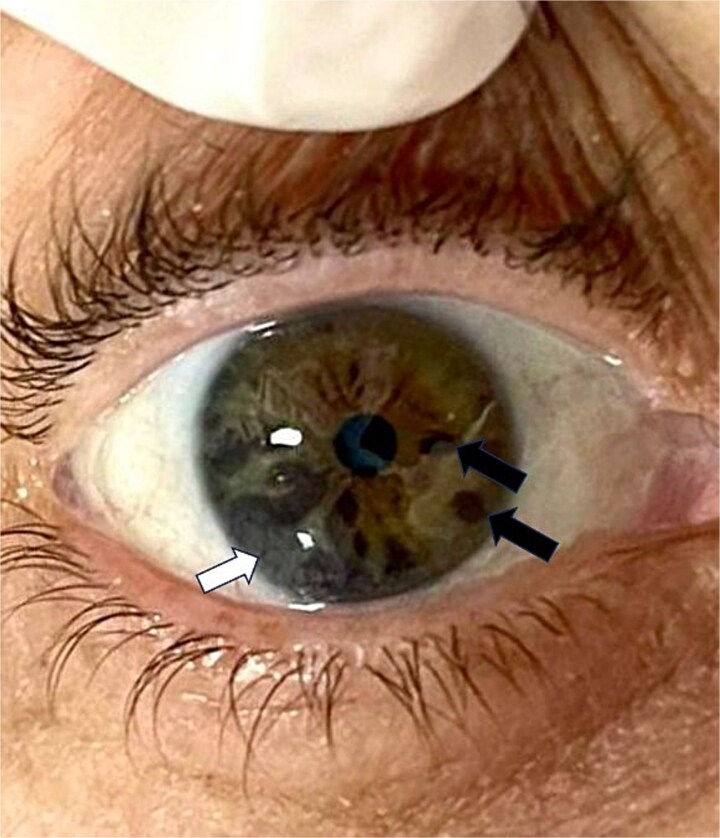
Iris metastatic melanoma lesion. Black-colored raised lesion located at the mid-inferior quadrant of the iris (white arrow), along with pigmented iris nevus (black arrows).

In January 2025, he presented to the emergency department with a one-month history of hyporexia, oral intolerance, recurrent vomiting, and 10-kg weight loss. Physical examination revealed abdominal distension, gastroalimentous emesis, tachycardia (137 beats/min), and hypotension (78/59 mmHg). Laboratory tests showed grade III anemia (Hb 6.47 g/dl) and metabolic acidosis with hyperlactatemia (lactate 7.1 mg/dl). Abdominal CT demonstrated jejunojejunal intussusception caused by an intraluminal mass with proximal small bowel dilatation, and other findings of tumoral progression (Fig. 3).

**Figure 3:**
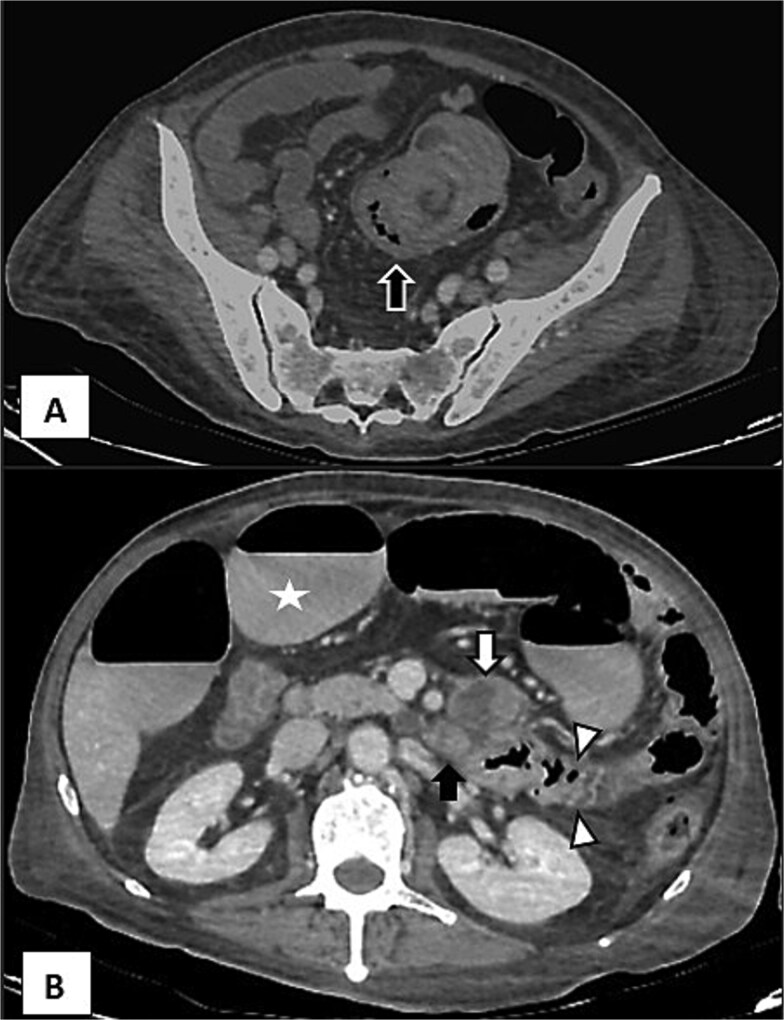
CT with venous phase showing intussusception and tumoral activity. (A) Intestinal intussusception showing “target sign” (black arrow) with wall oedema. (B) Upper view with distention of intestinal loops (white star) and tumoral activity adjacent to the third portion of duodenum (white arrow heads) associated to enlarged nodules (black arrow).

Exploratory laparotomy revealed marked jejunal distension and two endoluminal tumors (2.5 cm each) located 80 cm and 130 cm distal to the ligament of Treitz, respectively. Multiple firm nodules were palpated within the mesentery of the duodenum and jejunum, along with an additional tumor near the duodenum, adherent to the retroperitoneum (Fig. 4). A 60-cm segmental resection with 5-cm margins was performed using linear staplers, mesenteric dissection, and vascular ligation, followed by jejunostomy.

**Figure 4:**
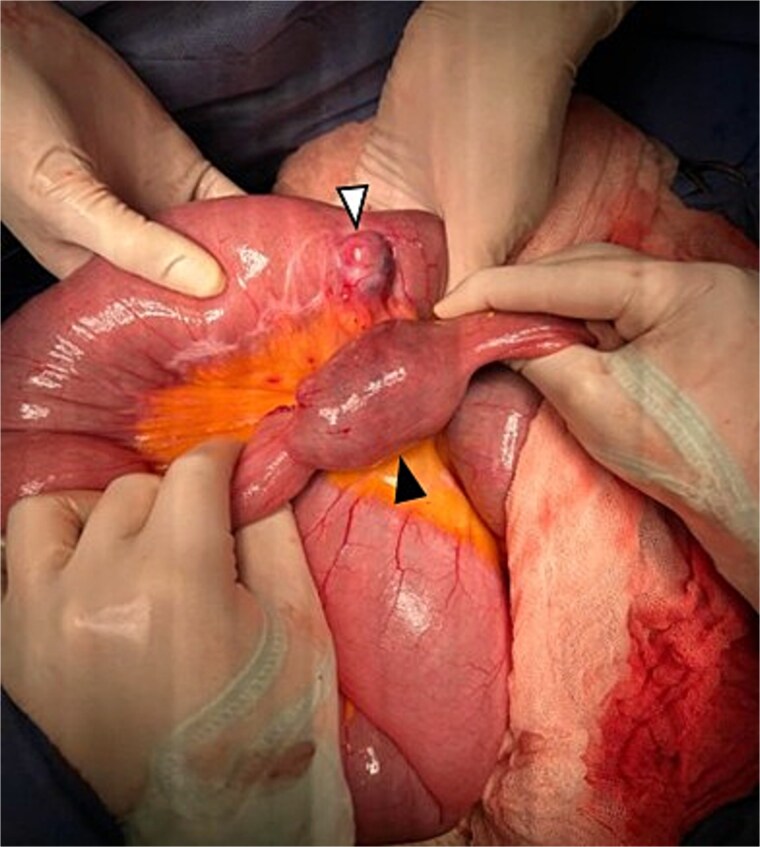
Surgical view showing two tumoral lesions depending from jejunum (white and black arrows). The largest lesion (black arrow) showing the site of intussusception.

In patients with GI metastases from MM, the disease typically represents an end-stage oncologic situation. In our patient, the presence of hemodynamic instability requiring vasopressor support, severe anemia, nutritional compromise manifested by sarcopenia and metabolic derangement, impaired oncologic status (ECOG 3, Karnofsky 50), and advanced age significantly reduced surgical tolerance with the risk of jejuno-ileal anastomotic leakage unacceptably high. Therefore, a jejunostomy was performed as a damage-control and palliative procedure, allowing resolution of the surgical emergency and restoration of enteral tolerance. Histopathological examination confirmed metastatic melanoma (Fig. 5). The postoperative course was uneventful, with adequate jejunostomy output (500–600 mL/day), and the patient was discharged on postoperative day eight with clinical improvement. However, progressive systemic disease with extensive mesenteric and pulmonary metastatic involvement led to subsequent respiratory failure and death two months later, reflecting tumor progression rather than a surgical complication. Regarding the expected outcomes and limitation in such cases, published surgical and oncologic series have shown that once melanoma has progressed to unresectable visceral metastatic disease, median survival is typically measured in months despite systemic therapy. In this context, reported median survival often ranges within only a few months [10], reflecting the aggressive biology and limited effectiveness of available treatments in end-stage disease. The two-month survival observed in our patient is therefore consistent with the natural history described in the literature for patients with non-resectable metastatic melanoma.

**Figure 5:**
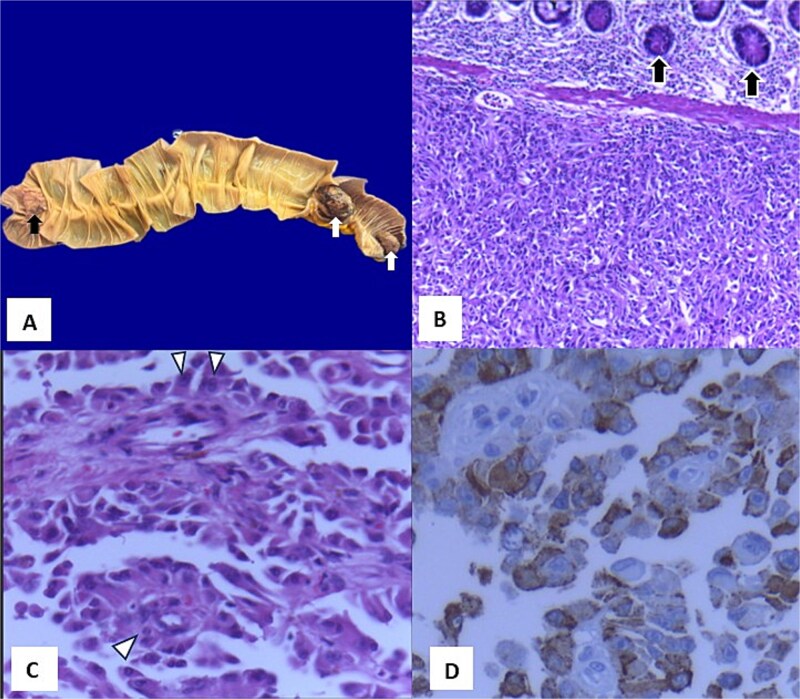
Histopathological analysis of the jejunal resected segment. (A) Macroscopic photograph of a small intestine segment showing an ulcerated lesion (black arrow) and two pedunculated nodular lesions (white arrows). (B) Panoramic photomicrograph (4x) stained with hematoxylin and eosin demonstrating replacement of the submucosa by a neoplasm composed of atypical epithelioid cells forming pseudorosettes (black arrows). (C) Photomicrograph at 40x magnification showing atypical epithelioid cells arranged around a fibrovascular core (white arrow heads). (D) Immunohistochemistry for Melan-a showing positive staining (brown color).

## Discussion

MM is an epithelial malignancy arising from melanocytes that originate from the neural crest [1, 8]. Melanocytes are found in various mucosal sites, including the eye, oral cavity, nasopharynx, anus, urinary tract, and vagina, although most melanomas occur in the skin [1]. Despite being the rarest form of skin cancer, melanoma accounts for approximately 1.7% of the global cancer diagnoses and is ranked as the fifth most common cancer in the United States in 2021—fifth in men and sixth in women [2, 8].

In Central and South America, the incidence of MM is approximately 1.4%, with acral lentiginous melanoma being the predominant subtype [3]. In Mexico, it represents the third most common skin cancer and accounts for up to 75% of skin cancer–related mortality, with the back being the most frequent site in men and the face in women [4].

GI metastases occur in up to 60% of advanced cases, but only 1%–5% are diagnosed antemortem [1, 5, 7, 8]. The small intestine is the most common site (51%–71%), followed by the stomach (27%), colon (22%), and esophagus (5%) [1, 5, 7, 8]. Within the small bowel, the jejunum and ileum are the most involved, possibly because of the high expression of CCR9 receptors interacting with CCL25 ligands in this region [1, 5]. The interval between the diagnosis of primary MM and GI metastasis ranges from 2 to 180 months [7].

The clinical presentation is often nonspecific, including abdominal pain, dyspepsia, vomiting, weight loss, obstruction (sometimes due to intussusception), perforation, or bleeding [1, 5]. Adult intussusception accounts for <5% of all cases, with 90% occurring secondary to an underlying pathology, most frequently in the small bowel [6]. Contrast-enhanced CT is the gold standard for diagnosis [6].

Histopathological confirmation is essential, allowing classification and molecular profiling (BRAF, PD-L1, and MEK) for therapeutic guidance. Surgical resection remains the cornerstone for symptom control or curative intent, showing superior 1- and 5-year survival rates compared with chemotherapy or immunotherapy alone [1, 9, 10]. Immunotherapy, particularly PD-1 inhibitors, immune checkpoint inhibitors, and targeted BRAF/MEK blockade, has markedly improved outcomes in selected cases [1, 10] but with adverse effects associated to myocarditis, pneumonitis or enteritis that can limit physiologic reserve.

This case highlights the importance of comprehensive staging and vigilance for rare GI manifestations of melanoma that may present as acute surgical emergencies, in which timely surgical intervention remains vital despite a poor overall prognosis. In elderly patients with advanced metastatic melanoma and severely deteriorated oncologic and physiological status, emergency surgical decision-making must be guided by risk stratification rather than technical feasibility alone. In our patient, the presence of hemodynamic instability requiring vasopressor support, severe anemia, poor performance status (ECOG ≥3), and diffuse mesenteric metastatic disease rendered the risk of jejuno-ileal anastomotic leakage unacceptably high. Although primary anastomosis is generally associated with lower morbidity in stable patients, in this context a palliative jejunostomy represented the safest and most appropriate strategy, providing rapid relief of obstruction and short-term improvement in quality of life. More broadly, high-risk features favoring stoma formation in this setting include hemodynamic instability, severe anemia or hypoalbuminemia, extensive peritoneal or mesenteric involvement, limited life expectancy, and compromised functional status, as primary anastomosis under these conditions confers no meaningful survival benefit while markedly increasing postoperative morbidity and mortality.
